# Exercise Capacity and Pulmonary Function in Pediatric Patients With Anomalous Pulmonary Venous Connection Post‐Surgical Repair: A Retrospective Analysis

**DOI:** 10.1002/kjm2.70101

**Published:** 2025-09-03

**Authors:** Yen‐Hsien Wu, Yen‐Sen Lu, Sheng‐Hui Tuan, Yi‐Ching Liu, I‐Ching Huang, Yi‐Cheng Wang, Tang‐Hsu Hsieh, Shih‐Hsing Lo, Ko‐Long Lin, Jong‐Hau Hsu

**Affiliations:** ^1^ Department of Pediatrics Kaohsiung Medical University Hospital, Kaohsiung Medical University Kaohsiung Taiwan; ^2^ Department of Physical Medicine and Rehabilitation Kaohsiung Medical University Hospital, Kaohsiung Medical University Kaohsiung Taiwan; ^3^ Institute of Allied Health Sciences, College of Medicine, National Cheng Kung University Tainan City Taiwan; ^4^ Department of Rehabilitation Medicine Cishan Hospital, Ministry of Health and Welfare Kaohsiung Taiwan; ^5^ Department of Physical Medicine and Rehabilitation, Faculty of Medicine, College of Medicine Kaohsiung Medical University Kaohsiung Taiwan; ^6^ School of Medicine, College of Medicine Kaohsiung Medical University Kaohsiung Taiwan

**Keywords:** anomalous pulmonary venous connection, exercise capacity, oxygen consumption

## Abstract

Anomalous pulmonary venous connection (APVC), including total (TAPVC) and partial (PAPVC) forms, is a congenital heart defect with abnormal pulmonary vein drainage; and while surgical repair has improved survival, its long‐term impact on cardiopulmonary function remains unclear. This retrospective study evaluated exercise capacity and pulmonary function in 26 pediatric APVC patients (17 TAPVC, 9 PAPVC) using cardiopulmonary exercise testing (CPET) and compared them with 63 age‐matched healthy controls. Patients with complex defects or significant comorbidities were excluded. Results showed significantly lower anaerobic threshold VO_2_ (*p* = 0.03), peak VO_2_ (*p* < 0.001) and peak heart rate (*p* = 0.02) in the APVC group, indicating impaired exercise capacity; though no differences were found between TAPVC and PAPVC subgroups. Despite preserved resting lung function, these findings suggest that children with repaired APVC experience persistent exercise limitations, underscoring the importance of routine functional assessment and potential rehabilitation, with further studies needed to clarify underlying mechanisms and guide long‐term care.

## 1 Introduction

Anomalous pulmonary venous connection (APVC) encompasses a spectrum of congenital heart defects characterized by abnormal connections between the pulmonary veins and the systemic venous circulation, and can be divided into total APVC (TAPVC) and partial APVC. While surgical repair has significantly improved the long‐term outcomes for patients with APVC [1], there remains a paucity of data regarding the impact of these anomalies on exercise capacity and cardiopulmonary function in children and adolescents post‐repair [2]. Understanding the functional implications of APVC is crucial for optimizing patient care and long‐term outcomes in this population.

Cardiopulmonary exercise testing (CPET) is a diagnostic tool used to evaluate the integrated function of the cardiovascular, pulmonary, and musculoskeletal systems during physical exertion [3]. By measuring parameters such as oxygen consumption (VO_2_), carbon dioxide production (VCO_2_), and ventilatory responses, CPET provides comprehensive insights into an individual's exercise capacity, aerobic fitness, and overall cardiopulmonary function [4]. This test involves monitoring a patient's physiological responses to incremental exercise on a treadmill or cycle ergometer, allowing for the assessment of both aerobic and anaerobic thresholds.

CPET is a valuable tool in diagnosing and managing patients with congenital heart defects (CHD), as it helps identify exercise intolerance and guides tailored therapeutic interventions. The 2025 AHA/ACC Guideline for the Management of Adults with Congenital Heart Disease emphasizes the importance of CPET in the evaluation and management of these patients [5, 6]. Its ability to offer detailed information on exercise performance and physiological responses makes it an essential tool for clinicians aiming to optimize patient care and improve quality of life through targeted treatments and rehabilitation strategies. Children with CHD, including those with APVC, face an elevated risk of mortality, particularly as they transition into later childhood and adolescence [7], with this increased mortality risk often being linked to complications related to impaired exercise capacity and cardiovascular function [8].

In this study, we aimed to assess the exercise capacity and resting pulmonary function of pediatric patients with APVC after surgical repair in the recent decade. By utilizing CPET and resting lung function tests, we sought to characterize the functional status of these patients and identify potential impairments in exercise tolerance and pulmonary function. Through comparison with a control group of healthy individuals, we aimed to elucidate the unique challenges faced by patients with APVC and inform clinical management strategies tailored to their needs.

## 2 Methods

We retrospectively analyzed data from pediatric patients with APVC who underwent CPET between 2005 and 2023 at two medical centers. Patients were included if they had APVC after corrective surgical repair. All patients underwent CPET at least 1 year after surgery to ensure complete physical recovery and were clinically stable to perform maximal CPET. Patients were excluded if they had concomitant CHD, residual lesions, or hemodynamic derangement, including significant pulmonary vein stenosis or pulmonary hypertension. A control group of age‐matched healthy individuals without known cardiopulmonary conditions was included for comparison. The study was approved by the Institutional Review Board of the Kaohsiung Medical University Hospital (number: KMUHIRB‐E(I)‐20240166).

Clinical data, including demographics, surgical history, and baseline characteristics, were collected from electronic medical records. All the recruited participants received symptom‐limited exercise testing using a treadmill‐based system equipped with a flow module, gas analyzer, and electrocardiographic monitor (Metamax 3B; Cortex Biophysik GmbH Co., Leipzig, Germany) to assess participants' exercise capacity. An experienced physiatrist with over 15 years of expertise in CPET (K.‐L.L.) supervised the entire procedure. Before the treadmill test, each participant was introduced to the procedures and equipment through a demonstration to ensure familiarity. All participants underwent exercise testing following the Bruce ramp protocol, as recommended by the American College of Sports Medicine (ACSM). The test was terminated when participants exhibited intolerable symptoms, were unable to continue, or reached maximal effort based on ACSM criteria [9]. Oxygen consumption (VO_2_) and carbon dioxide production (VCO_2_) were measured using a breath‐by‐breath method; additionally, blood pressure (BP), heart rate (HR) and respiratory exchange ratio (RER) were continuously monitored throughout the test, with oxygen consumption at AT (AT VO_2_) and oxygen uptake at maximal exertion (peak VO_2_) also being determined. The measured VO_2_ was divided by a constant (3.5 mL/kg/min) to calculate metabolic equivalents (METs); AT was identified using the ventilatory equivalents for oxygen (VE/VO_2_) and carbon dioxide (VE/VCO_2_) methods [10], while Peak VO_2_ was defined as the percentage of the measured peak VO_2_ to the predicted peak VO_2_ after comparing with the normal standards for cardiopulmonary responses to exercise in Taiwan [11].

The pulmonary function at rest was measured by using spirometry, including forced vital capacity (FVC), forced expiratory volume in one second (FEV_1_), and maximal voluntary ventilation (MVV). To evaluate lung function relative to predicted values, we calculated the ratios of measured to predicted values for FVC (FVC/FVCP), FEV_1_ (FEV_1_/FEV_1_P), and MVV (MVV/MVVP). Predicted values for each spirometry parameter were derived using reference equations specific to healthy children and adolescents in Taiwan [12].

All statistical analyses were performed using SPSS for Windows version 20.0 (Released 2011; IBM Corp, Armonk, NY). Continuous variables were summarized as mean ± standard deviation, while categorical variables were presented as absolute counts or percentages. The Kolmogorov–Smirnov test was employed to assess the normality of continuous data. To compare baseline characteristics and cardiopulmonary exercise parameters between the study and control groups, one‐way analysis of variance (ANOVA) with post hoc Scheffe's test was used for continuous variables that were normally distributed. For continuous variables that did not follow a normal distribution, the Kruskal–Wallis test with post hoc Dunn's test was applied, while categorical variables were compared using the Chi‐square test. Statistical significance was defined as a two‐sided alpha level of 0.05, with 95% confidence intervals reported.

## 3 Results

### 3.1 Patient Characteristics

A total of 26 APVC patients (mean age 18.61 ± 7.13 years at CPET; 17 TAPVC, 9 PAPVC post‐repair) and 63 age‐matched healthy controls (mean age 17.17 ± 8.12 years) were analyzed. Baseline characteristics did not differ significantly between groups (Table 1). Among APVC groups, all TAPVC patients received surgical repair in their early childhood, whereas PAPVC patients underwent surgery following symptom onset.

**TABLE 1:** Demographic characteristics.

	APVR (*N* = 26)	Control (*N* = 63)	*p*
Sex‐no. (%)			
Male	5 (23.8)	22 (34.9)	0.35
Female	16 (76.2)	41 (65.1)	0.47
Age (years)	18.61 ± 7.13	17.17 ± 8.12	
BH (cm)	153.77 ± 14.03	157.73 ± 12.94	0.24
BW (Kg)	48.16 ± 11.21	50.73 ± 15.56	0.49
BMI (Kg/m^2^)	20.06 ± 2.78	20.06 ± 4.05	0.99
Body fat	20.89 ± 7.65	20.85 ± 6.98	0.98
Resting SBP (mmHg)	119.81 ± 19.07	113.86 ± 12.07	0.10
Resting DBP (mmHg)	70.95 ± 8.97	68.90 ± 8.56	0.35
Resting HR (bpm)	79.90 ± 12.80	82.51 ± 12.78	0.42

Abbreviations: BH, body height; BMI, body mass index; BW, body weight; DBP, diastolic blood pressure; HR, heart rate; SBP, systolic blood pressure.

### 3.2 Resting Lung Function Test

Resting lung function test results for the control and APVC groups are presented in Table 2. Although all parameters including FVC, FEV_1_, FEV_1_/FVC, and MVV were lower in the APVC group, none met the clinical criteria for restrictive or obstructive lung disease; nor were significant differences of other parameters concerning resting lung function observed between the two groups.

**TABLE 2:** Performance of resting pulmonary function test of APVR and control groups.

	APVR (*N* = 26)	Control (*N* = 63)	*p*
FVC (L)	2.53 ± 0.81	2.87 ± 1.07	0.23
FVCP (%)	88.71 ± 21.38	95.42 ± 18.44	0.20
FEV1 (L)	2.14 ± 0.79	3.24 ± 0.71	0.34
FEV1P (%)	87.28 ± 25.5	94.17 ± 18.41	0.21
FEV1/FVC (%)	85.49 ± 12.18	88.25 ± 7.80	0.26
MVV (L/min)	57.98 ± 22.91	67.10 ± 29.54	0.43
MVVP (%)	84.21 ± 24.66	83.26 ± 46.62	0.96

Abbreviations: FEV1, forced expiratory volume in 1 s; FVC, forced vital capacity; FVCP, forced vital capacity percentage; MVV, Maximal voluntary ventilation.

### 3.3 Cardiopulmonary Exercise Testing (CPET)

Based on the exercise test data, the APVC group had significantly lower AT VO_2_ (20.92 ± 4.57 vs. 23.62 ± 5.08 mL/kg/min, *p* = 0.03) and peak VO_2_ (29.27 ± 7.14 vs. 35.57 ± 6.70 mL/kg/min, *p* < 0.001) values than the control group. Peak heart rate (169.33 ± 15.41 vs. 176.41 ± 10.98 bpm, *p* = 0.02) and peak PD value (75.40 ± 12.42 vs. 91.11% ± 10.67%, *p* < 0.001) were also significantly lower in the APVC group; although peak RER values were also lower in the APVC group, they remained above 1.1 in both groups (Table 3).

**TABLE 3:** Performance of exercise test of APVR and control groups.

	APVR (*N* = 26)	Control (*N* = 63)	*p*
AT VO_2_ (mL/kg/min)	20.92 ± 4.57	23.62 ± 5.08	0.03
AT HR (bpm)	137.19 ± 17.77	139.11 ± 13.53	0.61
PEAK VO_2_ (mL/kg/min)	29.27 ± 7.14	35.57 ± 6.70	< 0.001*
PEAK HR (bpm)	169.33 ± 15.41	176.41 ± 10.98	0.02
PEAK PD (%)	75.40 ± 12.42	91.11 ± 10.67	< 0.001*
PEAK VE (L/min)	45.89 ± 13.02	48.85 ± 15.58	0.44
PEAK RER	1.13 ± 0.07	1.18 ± 0.10	0.03*
PEAK SBP (mmHg)	153.35 ± 23.99	161.60 ± 31.55	0.29
PEAK DBP (mmHg)	76.25 ± 17.96	84.14 ± 18.57	0.09*

Abbreviations: AT, anaerobic threshold; DBP, diastolic blood pressure; HR, heart rate; PD, predicted value; RER, respiratory exchange ratio; SBP, systolic blood pressure; VE, minute ventilation; VO_2_, oxygen consumption.

* *p* < 0.05.

As for the comparison of CPET parameters between the TAPVC and the PAPVC groups, no significant differences were revealed in any of the measured parameters, including AT VO_2_ (21.24 ± 4.68 vs. 19.88 ± 4.52, *p* = 0.48), peak VO_2_ (29.60 ± 6.87 vs. 28.21 ± 8.69, *p* = 0.46), peak HR (171.19 ± 14.71 vs. 163.4 ± 17.84, *p* = 0.39) and peak PD (76.35 ± 13.66 vs. 72.35 ± 7.49, *p* = 0.56). These findings indicate no statistically significant differences in aerobic capacity or peak exercise performance between the two groups (Table 4).

**TABLE 4:** Comparison of total and partial anomalous pulmonary venous return.

	TAPVC (*N* = 17)	PAPVC (*N* = 9)	*p*
AT VO_2_ (mL/kg/min)	21.24 ± 4.68	19.88 ± 4.52	0.48
AT HR (bpm)	138.25 ± 17.73	133.8 ± 19.49	0.68
PEAK VO_2_ (mL/kg/min)	29.60 ± 6.87	28.21 ± 8.69	0.46
PEAK HR (bpm)	171.19 ± 14.71	163.4 ± 17.84	0.39
PEAK PD (%)	76.345 ± 13.66	72.35 ± 7.49	0.56
PEAK VE (L/min)	44.66 ± 13.7	49.8 ± 10.88	0.25
PEAK RER	1.14 ± 0.07	1.10 ± 0.03	0.38
PEAK SBP (mmHg)	153.87 ± 23.31	151.8 ± 17.58	0.99
PEAK DBP (mmHg)	78.6 ± 19.8	69.20 ± 8.73	0.54

Abbreviations: AT, anaerobic threshold; DBP, diastolic blood pressure; HR, heart rate; PD, predicted value; RER, respiratory exchange ratio; SBP, systolic blood pressure; VE, minute ventilation; VO_2_, oxygen consumption.

## 4 Discussion

Our study is the first to compare exercise performance in patients with APVC to that of an age‐matched healthy control group, with findings suggesting that children and adolescents with APVC following surgical repair exhibit reduced exercise capacity compared to their healthy peers, despite having preserved resting lung function.

Previous studies investigating exercise performance in patients with TAPVC mostly rely on predicted values due to a lack of exercise test results from normal healthy groups [13, 14]. Predicted values in CPET provide a standardized comparison for assessing cardiopulmonary function, aiding in diagnosis, risk stratification, and clinical decision‐making by adjusting for factors like age, sex, height, and weight. They are particularly useful for evaluating disease progression, treatment efficacy, and prognosis, especially in conditions like heart failure and pulmonary diseases; although their accuracy can be limited due to variability in reference equations, lack of representation for certain populations (e.g., athletes, elderly, and obese or chronically ill individuals) and failure to account for lifestyle factors such as training status and muscle efficiency. Additionally, some prediction models may overestimate or underestimate true physiological capacity and might not be fully applicable across different ethnic and regional groups, so while predicted values offer valuable insights, they should always be interpreted alongside actual patient data and clinical context to avoid misclassification and ensure accurate assessment [15]. In our study, we collected real CPET values from Asian individuals, which may help evaluate exercise performance in this population more accurately. By using region‐specific data, our findings could provide more precise reference values for assessing cardiopulmonary function, improving diagnostic accuracy and guiding clinical decision‐making for Asian populations.

The findings of our study contribute to the limited body of literature on the exercise capacity of pediatric patients with APVC following surgical repair. Our study reveals significant differences in exercise capacity between individuals with APVC and the control group. Despite no significant differences in baseline characteristics, individuals with APVC exhibited lower AT VO_2_ and peak VO_2_ values during CPET. Michael G. et al. reported that peak VO_2_ and percentage of predicted values were significantly lower than the normal values for age and gender [13]; similarly, the study by Paridon et al. found that AT VO_2_ among patients with APVC was below the mean value for healthy children of similar age and sex [14]. Their research reported chronotropic impairment in a small cohort (*N* = 9) of post‐surgical TAPVC patients, supporting our observation of reduced peak heart rate in the APVC group. They also suggest that anomalies in pulmonary venous connection, even after surgical repair, could contribute to reduced exercise tolerance in affected individuals.

Our study also identified significant differences in peak heart rate between individuals with APVC and healthy controls, consistent with the findings of Paridon et al. While the peak heart rate and VO_2_ difference is statistically significantly reduced in these patients, its clinical relevance lies in its potential impact on endurance and physical function during daily activities, particularly in school‐aged children, underscoring the importance of routine functional evaluation and potential early intervention in this population. Given the scarcity of exercise performance data for APVC patients and healthy controls since 2000, our findings indicate that impaired exercise capacity has persisted in APVC patients over the past two decades, despite advancements in surgical and medical management.

The mechanisms underlying the reduced exercise capacity observed in individuals with APVC are likely multifactorial. Hemodynamic abnormalities, such as pulmonary hypertension or ventricular dysfunction [16, 17], may contribute to impaired oxygen delivery and utilization during exercise [18]; additionally, alterations in pulmonary blood flow distribution and gas exchange might further compromise exercise tolerance in these patients [19, 20]. In our study, however, patients with pulmonary hypertension or pulmonary vein obstruction were excluded; yet a reduction in VO_2_ was still observed, and this might be related to subclinical cardiopulmonary limitations such as chronotropic incompetence or a sedentary lifestyle, as previously described in other reports [13, 21].

Our study found no significant impairment in resting lung function, as all lung function parameters were comparable to those of the control group. In 1993, Paridon et al. evaluated nine TAPVC patients after surgery, finding six of nine patients had a mild decrease in the values of FEV1 with a normal ratio of FEV1 to FVC [14]. Michael et al. also reported proportionate decreases in FVC and FEV1 with a normal FEV1/FVC ratio and proportionally greater reductions in mean expiratory flows (FEF25‐75), suggesting small airway dysfunction in their 27 TAPVC patients study in 2007 [13]. Our findings align with those of Paridon et al. and McBride et al.

In our study, we analyzed the results of both APVC patients and healthy individuals. Furthermore, our study expands upon these findings by including individuals with PAPVC after repair, providing additional insights into the impact of these anomalies on exercise capacity. PAPVC presents a complex challenge in both diagnosis and management due to its diverse clinical manifestations. Literature on PAPVC and CPET is limited [21], with our study being the first to present CPET findings in patients with PAPVC, indicating that patients with PAPVC after surgery also exhibit reduced exercise capacity compared to healthy controls. CPET parameters such as peak VO_2_ and AT VO_2_ are impaired in these patients, reflecting suboptimal cardiopulmonary function in these patients. However, the PAPVC subgroup included only nine patients, limiting the statistical power of subgroup analysis while reflecting the rarity of PAPVC cases and the additional difficulty of obtaining valid CPET data from pediatric patients.

Over the past decades, advancements in surgical techniques and equipment have improved surgical outcomes and survival rates for APVC repair [22]; although data on the long‐term cardiopulmonary outcomes following these advanced techniques remain limited. The identification of impaired exercise capacity in individuals with APVC has important clinical implications. Healthcare providers should consider incorporating exercise testing into the routine evaluation of these patients to assess functional status and guide treatment decisions. Tailored exercise rehabilitation programs might also be beneficial in improving exercise tolerance and quality of life in this population, while close monitoring for signs of exercise intolerance and cardiac decompensation is essential to optimize long‐term outcomes and prevent complications. In this retrospective study, we did not enroll these cases for the exercise rehabilitation program before CPET since they were clinically stable; although, owing to the results presented in the report, we organized this program for this select patient group and proposed CPET‐guided rehabilitation as a potential future direction.

This study has several limitations. First, participants were recruited from only two medical centers in Southern Taiwan, which might limit the generalizability of the findings; second, although no significant differences were observed in baseline characteristics between the control group and patients, confounding factors such as physical activity level, socioeconomic status, and nutrition were not assessed, which might have affected the observed differences in exercise capacity. Furthermore, the study was limited by its small sample size, retrospective design, and incomplete hemodynamic data; but given the rarity of APVC, our study represents the largest cohort to date. As a consequence, future multicenter studies incorporating more patients with comprehensive hemodynamic assessments and advanced imaging modalities are essential to better understand the pathophysiological mechanisms and to develop targeted interventions for improving exercise capacity in this population.

## 5 Conclusion

In conclusion, our study provides valuable insights into the exercise capacity of individuals with anomalous pulmonary venous connection. The observed impairments in exercise tolerance enhance the need for comprehensive evaluation and management strategies for this patient population.

## Conflicts of Interest

The authors declare no conflicts of interest.

## Data Availability

The data that support the findings of this study are available on request from the corresponding author. The data are not publicly available due to privacy or ethical restrictions.
